# A non-bilaterian perspective on the development and evolution of animal digestive systems

**DOI:** 10.1007/s00441-019-03075-x

**Published:** 2019-08-07

**Authors:** Patrick R. H. Steinmetz

**Affiliations:** Sars International Centre for Marine Molecular Biology, University of Bergen, Thormøhlensgt. 55, 5006 Bergen, Norway

**Keywords:** Cnidaria, Porifera, Placozoa, Ctenophora, Gastrovascular system, Gut evolution, Extracellular digestion, Intracellular digestion, Germ layer evolution

## Abstract

Digestive systems and extracellular digestion are key animal features, but their emergence during early animal evolution is currently poorly understood. As the last common ancestor of non-bilaterian animal groups (sponges, ctenophores, placozoans and cnidarians) dates back to the beginning of animal life, their study and comparison provides important insights into the early evolution of digestive systems and functions. Here, I have compiled an overview of the development and cell biology of digestive tissues in non-bilaterian animals. I will highlight the fundamental differences between extracellular and intracellular digestive processes, and how these are distributed among animals. Cnidarians (e.g. sea anemones, corals, jellyfish), the phylogenetic outgroup of bilaterians (e.g. vertebrates, flies, annelids), occupy a key position to reconstruct the evolution of bilaterian gut evolution. A major focus will therefore lie on the development and cell biology of digestive tissues in cnidarians, especially sea anemones, and how they compare to bilaterian gut tissues. In that context, I will also review how a recent study on the gastrula fate map of the sea anemone *Nematostella vectensis* challenges our long-standing conceptions on the evolution of cnidarian and bilaterian germ layers and guts.

## Introduction

Nutrient availability is rate limiting for most metabolic and cellular processes such as cell growth, cell division, motility or sensory activities. Three major classes of organic compounds are necessary for the normal functioning of metabolic processes in animals: carbohydrates (or saccharides), lipids and proteins (Voet et al. 2016). Carbohydrates are monomers or polymers (di-, oligo- or polysaccharides) of hexose sugar molecules (e.g. glucose, fructose or galactose) and comprise cellulose, starch (both glucose polymers) and chitin (*N*-acetylglucosamine polymer). Lipids are important for energy storage and production, and membrane formation. Triacylglycerides are the most common animal storage lipid, consisting of three fatty acids linked to a glycerol molecule by ester bonds. Proteins are polymers of amino acids, and crucial for almost all enzymatic activity (Voet et al. 2016).

**Fig. 1:**
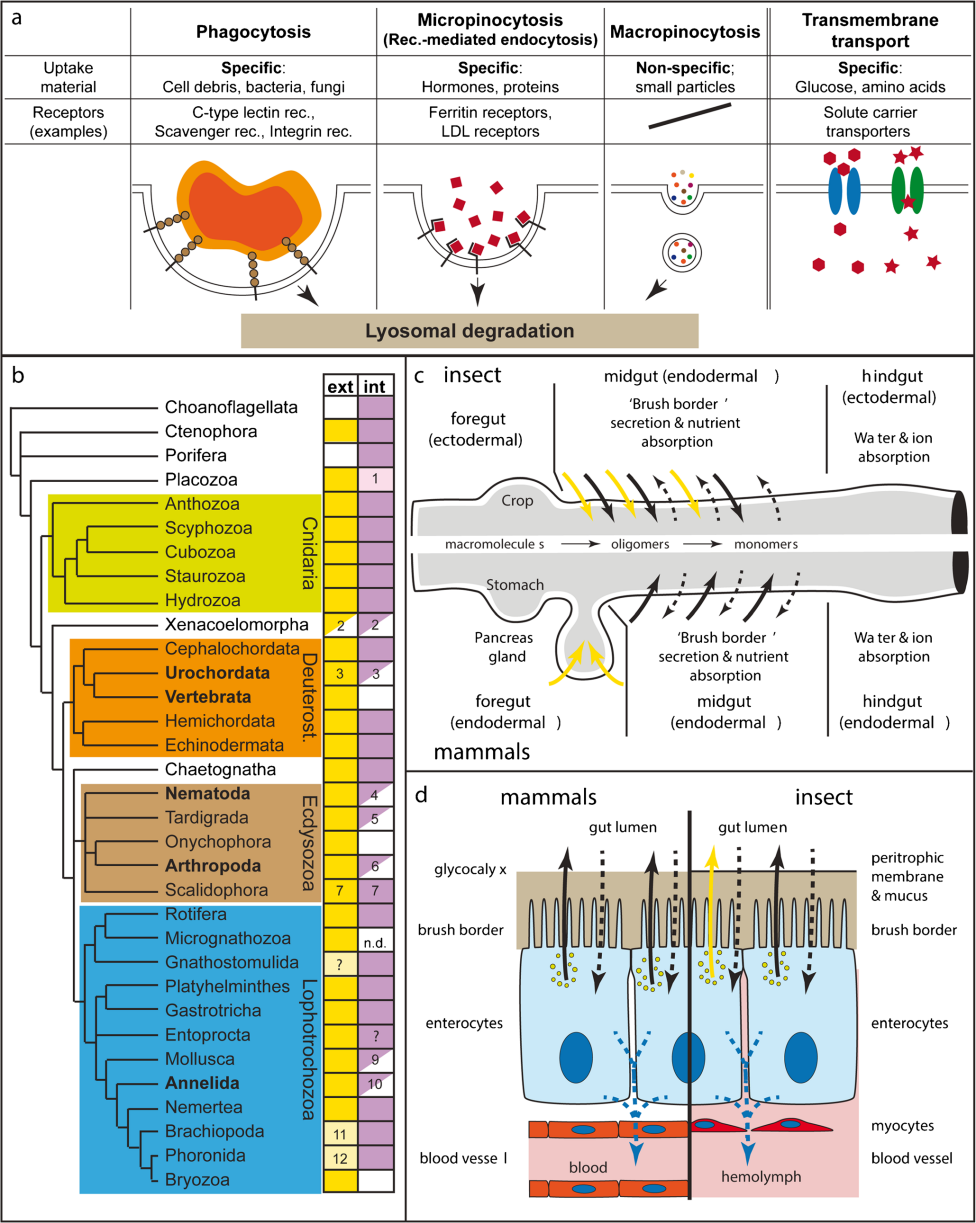
The molecular, cellular and phylogenetic basis of extracellular and intracellular digestion. **a** The basic molecular and cellular principles of nutrient ‘absorption’ by phagocytosis, receptor-mediated endocytosis, macropinocytosis or transmembrane transport. **b** The phylogenetic distribution of extracellular (‘ext’, yellow) and intracellular (‘int’, purple) digestion activities. White, empty boxes: absence of activity. Half boxes: partial absence of phagocytosis or of intracellular digestion in certain subgroups. Light colours represent minor roles of respective mode. Comments: (1) Uptake of ferritin suggests micropinocytosis; phagocytosis may be present in fibre cells, where an ‘immunity role’ is probable. (2) *Xenoturbella*: probably only macropinocytosis present (Israelsson 2008). (3) Secretion of lysosomal enzymes in *Ciona intestinalis* (Thomas 1970) and no phagocytosis, but only pinocytosis of HRP in *Oikopleura* (Cima et al. 2002). (4) Some nematodes show pinocytosis, but no phagocytosis. (5) Pinocytosis shown but no phagocytosis. (6) Pycnogonids, arachnids and crustaceans have both extra- and intracellular digestion. All other arthropod groups (with some exceptions, such as blood-sucking insects) lack intracellular digestion. (7) Present in Priapulida; in Kinorhyncha, Loricifera: extracellular digestion is likely, considering the presence of gland and zymogen cells; intracellular digestion is unstudied. (9) Cephalopoda present only pinocytosis. (10) Extracellular digestion dominates in most annelids, but intracellular digestion plays a role in leeches. In some polychaetes, phagocytic coelomocytes appear to invade the gut epithelium. (11) Minor role for extracellular digestion; only carbohydrase activity found. (12) Minor role for extracellular digestion. **c** Comparison between the insect (upper half) and mammal guts (lower half), and their secretory and absorptive capacities. Yellow arrows: secretion of polysaccharidase (e.g. Amylase) and endopeptidase (e.g. Trypsin). Black arrows: secretion of oligo- and disaccharidases, and oligo- and dipeptidases. Dashed arrows: Apical (black) and baso-lateral (blue) transmembrane transport of amino acids and monomeric carbohydrates (e.g. glucose, fructose).**d** Schematic representations comparing vertebrate and insect enterocytes. Colours as in (c). **b** is based on following, non-exhaustive list of references: Choanoflagellates (Dayel and King 2014), Ctenophora (Bumann and Puls 1997; Hernandez-Nicaise 1991), Porifera (Imsiecke 1993; Leys and Eerkes-Medrano 2006; Weissenfels 1982; Willenz and Van De Vyver 1982), Placozoa (Grell and Ruthmann 1991; Smith et al. 2014), Cnidaria (Arai 1997; Bouillon et al. 2006; Van-Praët 1985), Xenacoelomorpha (Israelsson 2008; Markosova 1986; Pedersen 1964), Cephalochordata (Biuw and Hulting 1971), Urochordata (Thomas 1970; Yonge 1937), Vertebrata (Karasov and Hume 1997), Hemichordata (Bridges and Woodwick 1994), Echinodermata (Tokin and Filimonova 1977), Chaetognatha (Arnaud et al. 1996), Nematoda (Clokey and Jacobson 1986; Riley 1973; Wright 1991), Tardigrada (Biserova and Mustafina 2015; Dewel et al. 1993; Yonge 1937), Onychophora (Heatley 1936; Manton 1937; Storch and Ruhberg 1993), Arthropoda (Ceccaldi 1989; Fahrenbach and Arango 2007; Filimonova 2008; Miguel-Aliaga et al. 2018; Wägele et al. 1981), Scalidophora (Kristensen 1991; Kristensen and Higgins 1991; Storch 1991; Storch et al. 1989), Rotifera (Wurdak 1987; Yonge 1937), Micrognathozoa (Møbjerg Kristensen and Funch 2000; Yonge 1937), Gnathostomulida (Lammert 1991),Platyhelminthes (Antoniazzi and Silveira 1996; Bowen et al. 1974; Jennings 1968; Ruppert et al. 2004), Gastrotricha (Ruppert 1991; Ruppert et al. 2004; Teuchert 1977), Entoprocta (Morton 1960; Ruppert et al. 2004), Mollusca (Boucaud-Camou and Yim 1980; Lobo-da-Cunha 2000; Owen 1974; Yonge 1937), Annelida (Jennings and Van Der Lande 1967; Jeuniaux 1969; Michel et al. 1984; Yonge 1937), Nemertea (Ruppert et al. 2004), Brachiopoda (Morton 1960; Steele-Petrovic 1976; Yonge 1937), Phoronida (Vandermeulen and Reid 1969), Bryozoa (Yonge 1937)

Eukaryotic cells take up nutrients either by transmembrane transport or vesicle-mediated uptake (endocytosis) (Fig. 1a) (Alberts et al. 2016). Transmembrane nutrient uptake from the environment typically involves transporters for amino acids or sugar monomers, which are shared between animals, plants and fungi (Wilson-O'Brien et al. 2010; Wipf et al. 2002). These are used in gut enterocytes as well as peripheral tissues (e.g. muscles, liver, neurons) of many bilaterian animals to take up nutrients from the digestive tube or blood stream.

Food particles or larger molecules (e.g. proteins, polysaccharides) cannot be taken up directly through the membrane but necessitate vesicle-mediated uptake. Depending on particle or molecule size, these uptake processes are subdivided into two broad classes, together called endocytosis: phagocytosis and pinocytosis. Phagocytosis leads to the engulfment and uptake of large particles (> 0.5 μm, e.g. bacteria, large organic debris). In bilaterians, it is best known for its role during innate immunity (Bayne 1990; Rosales and Uribe-Querol 2017). Particles are detected by typically low-specificity receptors (e.g. C-type lectins, scavenger receptors or integrins) that recognize surface molecules such as carbohydrates (e.g. mannose) of potential prey or pathogens (e.g. bacteria, fungi, algal cells) (Lancaster et al. 2019; Rämet et al. 2001; Rosales and Uribe-Querol 2017; Wang et al. 2014). Phagocytosis leads to the encapsulation of the particle in intracellular vesicles (‘phagosomes’) that will fuse with lysosomal vesicles for intracellular digestion (Rosales and Uribe-Querol 2017) (see also Hartenstein et al. this issue).

The subclasses of pinocytosis most relevant for nutritive purposes are micropinocytosis (which also includes receptor-mediated endocytosis) and macropinocytosis (independent of specific receptor-ligand interactions) (Fig. 1a; see also Hartenstein et al. in this issue). The result of these processes is the formation of an intracellular vesicle that will subsequently fuse with a digestive lysosome, which contains hydrolytic enzymes optimized to work at low pH levels (pH 4.0–4.5). Phagocytosis, macro- and micropinocytosis are thus all commonly classified as ‘intracellular digestion’ processes.

**Fig. 2:**
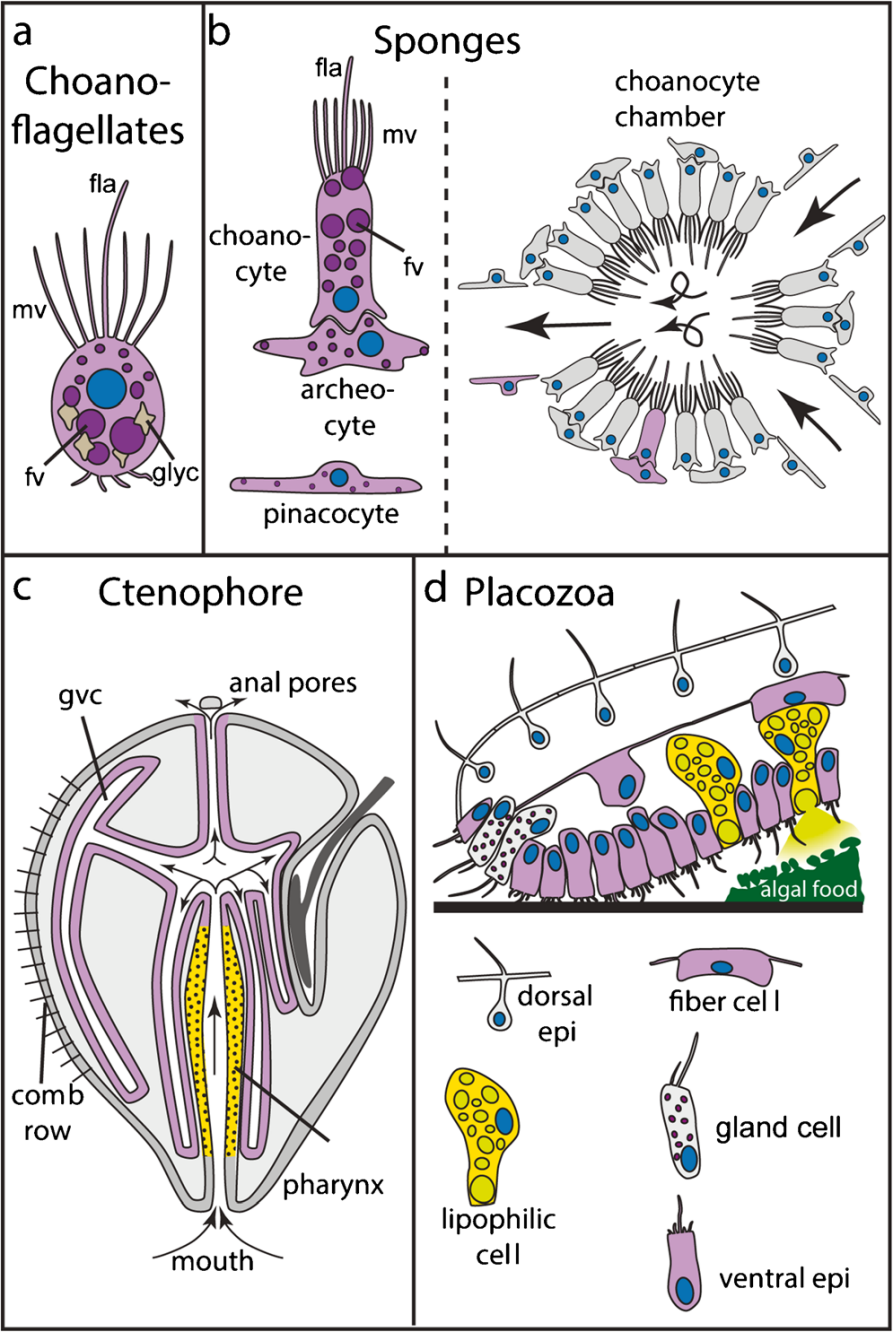
Digestive cell types and tissues of choanoflagellates (**a**), sponges (**b**), ctenophores (**c**) and placozoans (**d**). Purple cells represent cell types or tissues involved in phagocytosis or pinocytosis. Yellow cells/tissues: exocrine. Fla: flagellum; epi: epithelial cell; fv: food vesicle; glyc: glycogen particles; gvc: gastrovascular cavity; mv: microvilli

Choanoflagellates, ichthyosporeans and filastereans are protists, which are closely related to metazoans, and which mainly feed on bacteria by phagocytosis (Figs. 1b and 2a) (Dayel and King 2014; Sebe-Pedros et al. 2017). As phagocytosis is also the prevalent or only feeding mechanism in sponges, ctenophores, cnidarians and a subset of bilaterian animals (Fig. 1b; and below), it is most likely the ancestral feeding mechanism of metazoans (Lancaster et al. 2019). Notably, in a number of bilaterian groups including vertebrates, tunicates or insects, phagocytosis (and to a lesser extent pinocytosis) is mainly restricted to immune cells of the blood or nervous system and plays little or no role during intestinal digestion (Fig. 1b and references)(Lancaster et al. 2019; O'hagan 1996; Schafer and Stevens 2013; Yonge 1937). In these animals, extracellular digestive processes are very efficient and result in the direct absorption of nutrient monomers by the gut epithelia using transmembrane transport. As phagocytosis plays no or only a minor part during digestive processes in nearly all major genetic research organisms, the molecular and cellular basis of phagocytosis in bilaterian digestion remains largely understudied (see also Hartenstein et al. in this issue). It also remains unclear how gut cells using phagocytosis for food particle uptake can establish and maintain a stable gut microbiome.

Receptor-mediated endocytosis, present in all eukaryotic cells, has a wide range of cellular functions, including membrane protein recycling and nutrient uptake (Fig. 1a) (McMahon and Boucrot 2011). It plays a major role in lipid uptake through the recognition of lipoproteins by low-density lipoprotein receptors (LDLRs), or the uptake of iron-binding ferritin using ferritin receptors (McMahon and Boucrot 2011). In many animals, receptor-mediated endocytosis is important for transporting yolk components into the growing egg during oogenesis (Wourms 1987).

The emergence of extracellular digestion is considered a major step during animal evolution (Nielsen 2008). The evolution of extracellular digestion has been extraordinary beneficial in many ways (Yonge 1937): (i) The size of particles taken up by phagocytosis is limited by the size of the phagocytic cell. Extracellular digestion overcomes these limitations by breaking up larger pieces of food into smaller particles. Its evolution has thus allowed animals to feed on large organisms and has widely expanded available food resources. (ii) Intracellular digestion is limited by lysosome availability. A higher rate of intracellular digestion is thus only made possible by a rise in cell numbers, which has led to an enlarged surface area of bilaterian gut regions with intracellular digestion. The spatial constraints of surface enlargement are overcome by extracellular digestion. (iii) The constant generation of new, single-use lysosomal vesicles for intracellular digestion is energetically costly due to the ATP-dependent acidification of the lysosomal content, and the synthesis of new digestive enzymes. As extracellular digestive enzymes can digest much larger food particles, it is possible that their extracellular use is more efficient in comparison to intracellular digestion by lysosomal enzymes.

Extracellular digestion in animals is always linked to the presence of a digestive cavity, which prevents the diffusion of enzymes and nutrients into the environment. A first concept of the evolution of digestive cavities and extracellular digestion dates back to almost 150 years and is tightly connected with the discovery of germ layers. Thomas Huxley, in his discovery of cnidarian endoderm, recognized similarities with vertebrate endoderm as both are involved in forming a digestive cavity (Huxley 1849). Based on the hypothetical common origin (homology) of cnidarian and bilaterian endoderm and digestive cavities, Ernst Haeckel proposed a scenario of animal evolution, his famous ‘gastraea theory’ (Haeckel 1873). It mainly states that endoderm is homologous among all animals, and that its formation during gastrulation recapitulates the early evolution of the primitive gut. Haeckel claims, therefore, that glandular and absorptive derivatives of the endoderm must also be homologous among all animals. This narrative, despite being almost 150 years old, is still the most popular scenario to explain the early evolution and development of animal body plans (Arendt et al. 2015; Brunet and King 2017; Nielsen 2008; Stainier 2005). Accordingly, it is a commonly accepted concept that a blind-ended gut evolved by the invagination of cells at one side of a blastula-like cell sphere during early metazoan evolution. This blind gut, corresponding to the digestive sac of extant cnidarians, is thought to have directly transformed into the through-gut of bilaterians, but how this transition has occurred is highly debated (Hejnol and Martindale 2009; Nielsen et al. 2018). Here, I will provide an overview of our current understanding of the development and cell type composition of non-bilaterian digestive tissues. Wherever possible, these data will be discussed in a broader evolutionary context and in the light of Haeckel’s gastraea theory. A particular focus will be put on a recent study of the gastrula fate map of a sea anemone, challenging the homology of the endodermal germ layer and digestive tissues between cnidarians and bilaterians. I will discuss an alternative scenario of germ layer evolution, and its profound consequences on our understanding of bilaterian gut and germ layer evolution.

## Development and cell type diversity of animal digestive systems

### Extracellular digestion and an efficient through-gut characterize most digestive systems in Bilateria

In order to provide a reference for evolutionary comparisons, it is necessary to give a short overview of the development and main cell types of digestive systems in bilaterian animals (e.g. vertebrates, insects, annelids, molluscs). All major bilaterian groups, with a few exceptions (e.g. platyhelmintes or xenacoelomorphs), possess a through-gut with an anterior mouth and a posterior anus (Brusca et al. 2016; Hejnol and Martin-Duran 2015). The bilaterian digestive tract is typically subdivided into an anterior foregut, a median midgut and a posterior hindgut (see also Hartenstein et al. in this issue). The bilaterian gut usually starts developing with the formation of germ layers during gastrulation. While fore- and hindgut develop from either endo- or ectoderm, the midgut consistently develops from the endodermal germ layer (Gilbert and Barresi 2016; Nowotschin et al. 2019). The midgut is typically glandular and absorptive (Fig. 1c), while the hindgut is re-uptaking water and ions (Fankboner 2003; Karasov and Hume 1997).

Extracellular digestive enzymes are predominantly secreted from exocrine cells in the endodermal midgut (or vertebrate endodermal foregut) and its associated glands (Fankboner 2003; Gilbert and Barresi 2016). Vertebrate digestive enzymes are secreted from pancreatic acinar cells (e.g. amylase, trypsin-like endopeptidases, pancreatic lipase) and small intestinal enterocytes (e.g. isomaltase, sucrase, dipeptidase). In hagfish, lampreys, cephalochordates and tunicates, pancreatic-like exocrine cells are interspersed among enterocytes of the gut (Biuw and Hulting 1971; Youson and Al-Mahrouki 1999), suggesting it is the ancestral situation in chordates. While pancreatic acinar cells exhibit a typical, vesicle-filled gland cell morphology, enterocytes are typically columnar epithelial cells with a large number of apical microvilli (‘brush border’) (Fig. 1d). Vertebrate enterocytes are bi-functional: they secrete digestive enzymes acting within the extracellular matrix covering of the brush border microvilli (‘glycocalyx’) and shuttle nutrients from the gut lumen to the circulatory blood system (Fig. 1d) (Hooton et al. 2015; Karasov and Hume 1997). The complementary cocktails of enzymes produced by the pancreatic exocrine cells and enterocytes continuously break down large macromolecules into monomers during the unidirectional transport of food along the digestive tract (Fig. 1c) (Karasov and Hume 1997). Pancreatic and brush border enzymes function optimally at neutral pH, a fundamental difference to lysosomal or gastric enzymes (e.g. gastric lipase, cathepsins) (Voet et al. 2016). Free amino acids or carbohydrate monomers then selectively cross the enterocyte membrane by transmembrane transport using ion-dependent or independent amino acid or carbohydrate transporters (e.g. GLUT glucose transporter) (Karasov and Hume 1997). Fatty acids and monoacylglycerides enter enterocytes either by diffusion or protein-mediated uptake (Hussain 2014).

In insects, midgut enterocytes also have dual exocrine and absorptive roles (Fig. 1d), and similar signalling pathways control their differentiation from intestinal stem cells in vertebrates and *Drosophila* (Apidianakis and Rahme 2011; Dutta et al. 2015). Enterocytes of *Drosophila* secrete a mix of digestive enzymes that, in contrast to vertebrates, can digest triacylglycerides and the whole range of sizes of proteins and carbohydrates from macromolecules to monomers (Miguel-Aliaga et al. 2018). In addition, they express transmembrane transporters for glucose and amino acids. The specific enzyme and transporter profiles of enterocytes strongly differ between gut regions (Dutta et al. 2015). Analogous to vertebrates, insect enterocytes shuttle nutrients from the gut lumen to the hemolymph in order to supply nutrients to the rest of the body (Miguel-Aliaga et al. 2018).

Notably, a number of deuterostomes (cephalochordates, hemichordates and echinoderms), lophotrochozoans (e.g. brachiopods, phoronids, platyhelminths) and ecdysozoans (e.g. tardigrades, scalidophors) use intracellular digestion by phago- or pinocytosis, often in combination with extracellular digestion (Fig. 1b). Currently, very little developmental or molecular data is known about phagocytic cells (phagocytes) in the guts of invertebrate bilaterians. It is thus also unclear if insect/vertebrate enterocytes (secretory and absorptive) and invertebrate gut phagocytes (mainly phagocytic) share common developmental or evolutionary origins (see also Hartenstein et al. in this issue).

### Sponges are efficient filter feeders that use almost exclusively intracellular digestion

Sponges (Porifera) are subdivided into four large groups: demosponges (e.g. *Amphimedon queenslandica*, *Ephydatia fluviatis*, *Tethya wilhema*, *Spongilla sp.*), calcareous sponges (e.g. *Sycon sp.*), glass sponges (Hexactinellida; e.g. *Aphrocallistes sp.*) and homoscleromorph sponges (e.g. *Oscarella* sp.). They are mostly marine filter feeders, but some molecular model species are freshwater sponges (e.g. *Ephydatia*, *Spongilla*). The cell biology and physiology of feeding has been mainly investigated in demosponges, comprising the majority of all sponge species, and calcareous sponges (Leys and Hill 2012). Sponges have long been considered to lack epithelia, but recent studies have revealed the presence of some typical epithelial components (cell polarity and cell junction genes), and a capacity to seal their internal space from the environment (Adams et al. 2010; Belahbib et al. 2018; Fahey and Degnan 2010; Leys and Hill 2012; Leys et al. 2009; Riesgo et al. 2014). This situation limits the diffusion of nutrients between the environment and the extracellular matrix (mesohyl). Sponges feed based on one of the most efficient water filtering systems in the animal kingdom: an intricate canal system of choanocyte chambers generates water flow through a multitude of inlet pores (Fig. 2b) (Harrison and de Vos 1991; Leys and Hill 2012). Choanocytes consist of cells with a single flagellum, whose beating creates a water flow, and an apical microvilli-based collar that serves as efficient filtering apparatus for bacteria and particles (Harrison and de Vos 1991; Laundon et al. 2019) (Fig. 2b). Based on bead or algae uptake experiments and ultrastructural evidence, they are the main phagocytic cell type and show intracellular digestion activity (Gonobobleva and Maldonado 2009; Imsiecke 1993; Laundon et al. 2019; Leys and Eerkes-Medrano 2006; Willenz and Van De Vyver 1984). Choanocytes develop from pluripotent archaeocytes, and together with those, they constitute the major stem cell types in sponges (Funayama 2013; Funayama et al. 2010).

Choanocytes share a number of ultrastructural similarities with choanoflagellate protists, such as the water flow-creating flagellum and a filtering ring of apical microvilli (Fig. 2a, b) (Laundon et al. 2019; Mah et al. 2014). Differences exist in the interaction between flagellum and microvilli, or the recently suggested lack of glycogen reserves in sponge choanocytes (Fig. 2a, b) (Laundon et al. 2019). In line with Haeckel’s gastraea theory, it has long been proposed that the similarities of both cell types are due to a common ancestry, and that the last common ancestor of all animals therefore consisted of a uniform, ball-like colony of choanocyte-like cells (Arendt et al. 2015; Brunet and King 2017; Cavalier-Smith 2017; Nielsen 2008). The controversial phylogenetic position of sponges and ctenophores could complicate this view in case that ctenophores, and not sponges, are confirmed as sister group to all remaining animals (Ryan et al. 2013; Simion et al. 2017; Whelan et al. 2015). Also, a recent single-cell transcriptomic study proposes that choanoflagellates are transcriptionally more similar to sponge archaeocytes (see below) than to choanocytes (Sogabe et al. 2019). These results raise the possibility that choanocytes are a more specialised sponge cell type than previously thought, and that the stem cell-like archaeocytes represent a more ancestral metazoan cell type.

Two other sponge cell types contribute to sponge feeding: pinacocytes that cover the outer and inner canal surfaces, and the totipotent archaeocytes localised in the intermediate mesohyl matrix (Fig. 2b). Pinacocytes have been proposed to collect, take up and intracellularly digest particles by phagocytosis, especially near water inlet pores (Frost 1976; Imsiecke 1993; Willenz and Van De Vyver 1982; Willenz and Van De Vyver 1984; Willenz et al. 1986). Archaeocytes (or amoebocytes) are mainly known for their role as stem cells but have also been described as potential nutrient transport cells from choanocytes to the rest of the body (Fig. 2b) (Frost 1976; Funayama 2010; Imsiecke 1993; Weissenfels 1982). Choanocytes and archaeocytes thereby combine three of the fundamental features of unicellular protists: nutrient uptake, intracellular digestion and cell division (De Goeij et al. 2009; Funayama 2010; Funayama et al. 2010; Willenz and Van De Vyver 1984). A single cell dataset for the demosponge *Amphimedon* has annotated clusters of ‘metacells’ based on published diagnostic cell type markers (Funayama et al. 2010; Sebé-Pedrós et al. 2018a). Interestingly, lysosomal enzymes (cathepsins, arylsulfatases, α-galactosidases) are over-represented mainly in choanocytes, while archaeocytes and pinacocytes only show over-representation of a subset of cathepsins (personal observations). Provided that the annotation of metacells is further confirmed by additional *in situ* hybridisation experiments, these observations highlight the importance of choanocytes as main digestive cell type and supports the previously observed role of intracellular digestion in pinaco- and archaeocytes.

Sponges are the only animal phylum where intracellular digestion is predominant. So far, digestive gland cells, found in all other animal groups, have not been described for sponges in the literature. A family of carnivorous, deep sea sponges (Cladorhizidae) shows however a highly specialised mode of potential extracellular digestion. Their members feed by digesting copepods, and lack a canal system and choanocyte chambers (Vacelet 2007; Vacelet and Boury-Esnault 1995; Vacelet and Duport 2004). They engulf their prey by pinacocytes and form a cyst where primary lysis occurs. Particles are subsequently phagocytosed and intracellularly digested by archaeocytes and so-called bacteriocytes. Prey decomposition times strongly suggest the activity of extracellular enzymes, but there is currently no direct evidence for their involvement, or for their potential bacterial or poriferan origin (Vacelet and Duport 2004). If further corroborated, this small group of deep sea sponges could represent a spectacular case of independent evolution of extracellular digestion in animals.

Due to the plasticity and diversity of the sponge body plan and modes of embryonic development (Ereskovsky 2010; Leys and Ereskovsky 2006), it is very difficult to draw comparisons between the digestive system and cell types of sponges and of cnidarians or bilaterians. Choanocytes are sometimes considered as ‘prototypical enterocytes’ and could be considered to form a primitive digestive cavity (Takashima et al. 2013). It is however difficult to infer from the present data that choanocytes and vertebrate/insect gut enterocytes share a common ancestry and are thus homologous cell types. Major differences between choanocytes and vertebrate/insect enterocytes exist: (i) choanocytes do not secrete digestive enzymes; (ii) choanocytes are not terminally differentiated, but pluripotent. They can give rise to sperm cells (Gaino et al. 1984; Paulus and Weissenfels 1986) and are able to divide and trans-differentiate into archaeocytes or pinacocytes during regeneration (Borisenko et al. 2015; Funayama 2010; Funayama et al. 2010; Sogabe et al. 2019). (iii) The known choanocyte gene expression profile (e.g. *piwi*, Wnt signalling genes, Sox genes, NK genes) has so far not revealed any similarities with bilaterian gut or enterocyte cell types (Fortunato et al. 2012; Funayama et al. 2010; Gazave et al. 2008; Leininger et al. 2014). In general, a better molecular and developmental understanding of choanocytes, cnidarian phagocytic cells present in the gastrodermis and bilaterian gut phagocytes is needed to infer if these cell types share a common ancestry or not.

### Ctenophores are an enigmatic, potentially ancient group of animals with sophisticated digestive systems

Ctenophores (‘comb jellies’) are predatory, gelatinous animals with superficial similarities to cnidarian jellyfish. Recent molecular phylogenies consistently group ctenophores as unrelated to cnidarians with a branching near the basis of the animal tree, but their definite position remains highly debated (Fig. 1b) (Dunn et al. 2008; Ryan et al. 2013; Simion et al. 2017; Whelan et al. 2015). They consist mainly of two epithelia with an intermediate extracellular matrix that harbours muscle, nerve and amoebocyte-like cells (Hernandez-Nicaise 1991). The mouth opens into a ciliated pharynx, abundant with gland cells, where extracellular digestion takes place (Fig. 2c) (Hernandez-Nicaise 1991).

The ctenophore digestive tract forms during gastrulation. Epibolic movement of the ectoderm leads to the internalisation of endodermal cells (Byrum and Martindale 2004). The exocrine pharynx is of ectodermal origin, while the phagocytic and intracellular digestive gastrovascular canal system develops from endoderm (Martindale and Henry 1999) (Hernandez-Nicaise 1991). This is very similar to the situation in sea anemones and octocorals, where the pharyngeal derivatives are ectodermal and constitute the main digestive exocrine tissue, while the endoderm forms the gastrovascular and intracellular digestive tissue (see below, (Ax 1996; Chia and Crawford 1973; Steinmetz et al. 2017; Wilson 1883; Wilson 1884). A number of developmental genes have been found expressed in the larval pharynx of *Mmemiopsis leidyi*, for example genes belonging to the TGFβ or Wnt signalling pathway, or a number of genes from the Sox, Tbx or Homeobox transcription factor families (Martindale and Henry 2015). For many of these transcription factors, it is difficult to determine their direct homologues in cnidarians or bilaterians. As typical pharynx marker genes of sea anemones (see below) are mostly absent (or not studied yet) in ctenophores, it remains currently unclear if the cnidarian and ctenophore exocrine pharynx share a common evolutionary origin.

In contrast to sea anemones, very little is known about pharyngeal cell types in ctenophores. A granular gland cell, proposed to have digestive functions, is abundant in the pharynx (Hernandez-Nicaise 1991). It contains strongly acidophilic vesicles with unknown content. Crude cell extracts (thus not distinguishing between lysosomal and secreted enzymes) have shown typical lysosomal enzyme activities, such as aminopeptidase, phosphatase or acidic chitinase (Hoeger and Mommsen 1984).The genome of *Mnemiopsis* contains *chitinase* genes, but not *pancreatic lipase* genes, which appear to have evolved in the last common ancestors of placozoans, cnidarians and bilaterians (Steinmetz et al. 2017). The pharyngeal digestive juice of *Mnemiopsis* is slightly acidic (pH 5–6.3) (Bumann and Puls 1997) and shows proteolyic activities in *Pleurobrachia* at acidic (ph 5.75) and neutral pH (pH 7.5) (Fankboner and Reid 1978). The responsible proteolytic enzymes have not been identified, but inhibitor and activator studies suggest activities of tryptic and cathepsin B-like proteases in *Pleurobrachia* (Fankboner and Reid 1978). The acidophilic and cathepsin-like nature of some enzymes implies that at least some of the extracellular digestive enzymes of the ‘granular gland cells’ are lysosomal-like. Based on these observations, it can be speculated that extracellular digestion in ctenophores has evolved by co-option of ‘exocytosis’, where lyososmal enzymes are secreted into an acidic extracellular environment, a process that appears widespread among mammalian cells (Appelqvist et al. 2013). At least a subset of ctenophore granular gland cells would thus be fundamentally different from cnidarian zymogen and bilaterian digestive exocrine cells, as the latter secrete enzymes active at neutral pH and show little or no resemblance to lysosomal enzymes.

Food debris is disintegrated by pharyngeal enzymes, further transported to the stomach and distributed through the meridional canal system (Fig. 2c) (Bumann and Puls 1997). Absorption of both food and ferritin particles into ciliated, epithelial cells of the canal system suggest that both phagocytosis and pinocytosis are active in these cells (Bumann and Puls 1997; Franc 1972; Presnell et al. 2016). It was recently re-confirmed that excretion of undigested particles in ctenophores occurs mainly through ‘anal pores’ opposite of the mouth (Agassiz 1850; Bumann and Puls 1997; Chun 1880; Main 1928; Presnell et al. 2016; Tamm 2019). The functional similarities between the through-guts of ctenophores and bilaterians raised speculations about their common ancestry, but the lack of diagnostic genes for cnidarian or bilaterian pharynx, midgut or anus (e.g. *foxA*, *hex*, *nkx2.1*, *evx*, *goosecoid*) in ctenophores makes a comparison of these regions on a molecular level currently difficult (Hejnol and Martin-Duran 2015; Steinmetz et al. 2017).

### Placozoans are ciliated, bi-layered discs with a gut-like lower epithelium

This phylum is represented by *Trichoplax adhaerens* and a few cryptic species, and most likely forms the phylogenetic sister group of cnidarians and bilaterians (Fig. 1b) (Osigus et al. 2019; Srivastava et al. 2008; Voigt et al. 2004). The very simple body plan of *Trichoplax adherens* consists of a flattened, ciliated disc with no axis of symmetry and is used to crawl on the substrate (Fig. 2d) (Grell and Ruthmann 1991; Schierwater 2005). It consists of an upper and lower epithelium without basal lamina, and a layer of intermediate, interconnected fibre cells (Fig. 2d) (Grell and Ruthmann 1991; Schierwater 2005). *T. adhaerens* feeds by crawling onto larger food particles (e.g. algae) and secretes digestive enzymes from the lower epithelium into a ‘digestive bag’ (Grell and Ruthmann 1991). The proposal of a homology between this primitive digestive cavity and the ‘archenteron’ of bilaterians and cnidarians goes back to Haeckel’s ‘gastraea’ theory, but has remained speculative ever since (Grell and Ruthmann 1991; Syed and Schierwater 2002). The process of nutrient uptake has been studied mainly on an ultrastructural level. Indirect evidence from feeding experiments indicates that micropinocytosis occurs in ciliated, non-glandular cells of the lower epithelium (Fig. 2d) (Ruthmann et al. 1986; Ruthmann and Terwelp 1979). Fibre cells in the middle layer contain starch granules and show potential phagocytic activity (Fig. 2d) (Grell and Ruthmann 1991). As so far, phagocytosis has not been observed in any cells of the outer epithelia; it remains unclear how larger particles enter the space between the upper and lower layer. Recently, the use of electron and fluorescent microscopy techniques has revealed novel insights into digestive cells types in *Trichoplax* (see also Smith et al., this issue). Notably, gland cells located at the rim, which have traditionally been connected to secretion of digestive enzymes, are proposed to have neurosecretory functions with roles in behavioural responses to food presence (Fig. 2d) (Senatore et al. 2017). Instead, newly identified ‘lipophil’ cells are characterized by a larger number of smaller vesicles, and a single large inclusion at the apical side with an acidic and lipophilic content (Fig. 2d) (Smith et al. 2015; Smith et al. 2014). Secretion of the large inclusion occurs during crawling of *Trichoplax* over algal food particles. This observation supports a potential role of ‘lipophilic cells’ in exocrine secretion of digestive enzymes (Fig. 2d) (Smith et al. 2015).

Ciliated ventral epithelial cells (‘cylinder cells’) are the second major cell type in the lower epithelium. The vesicular uptake of ferritin, the presence of coated vesicles and a large number of microvilli suggest that nutrient uptake occurs by receptor-mediated endocytosis in these cells (Grell and Ruthmann 1991). Transmembrane transport of amino acids or sugar molecules has to my knowledge so far not been tested. As embryonic stages beyond cleavage stages remain undescribed, the development of the digestive cell types, and potential similarities with cell types from other phyla remain enigmatic (Eitel et al. 2011; Grell and Ruthmann 1991). A recent single cell transcriptomic study has broadly confirmed the diversity of cell types, but as almost no expression data on cell type-specific genes is available to link the resulting ‘metacell’ clusters to the morphologically described cell types, their annotation is currently poorly supported (Sebé-Pedrós et al. 2018a). Validating the single-cell transcriptomic dataset using *in situ* hybridisation will provide a much more solid view on the molecular and functional diversity of the cell types in *Trichoplax*. It will be exciting to study how the expression profile (e.g. of transcription factors, digestive enzymes or metabolic transporters) of lipophilic, gland and ventral epithelia cells compare to bilaterian and cnidarian digestive cell types. Their study will have a large potential to shed light on the evolution and homology of digestive cells between placozoans, bilaterians and cnidarians.

### Cnidarians use a combination of extra- and intracellular digestion in a sac-like gastrovascular system

Cnidarians are the phylogenetic sister group to bilaterians and thus occupy an important position for the purposes of reconstructing the digestive system and cell types of the last common ancestor of cnidarians and bilaterians. Cnidarians are further subdivided into five large phylogenetic groups (Fig. 1b): anthozoans (sea anemones & corals; e.g. *Nematostella, Acropora*), scyphozoans (‘true jellyfish’, e.g. *Aurelia*), cubozoans (box jellyfish, e.g. *Tripedalia*), staurozoans (‘stalked jellyfish’, e.g. *Haliclystus*) and hydrozoans (e.g. *Hydra, Hydractinia*, *Clytia*). While anthozoans have only a sessile polyp stage, all other cnidarian groups have an additional pelagic medusa (jellyfish) life-stage. Coloniality is common among anthozoan, scyphozoan and hydrozoan polyps.

**Fig. 3:**
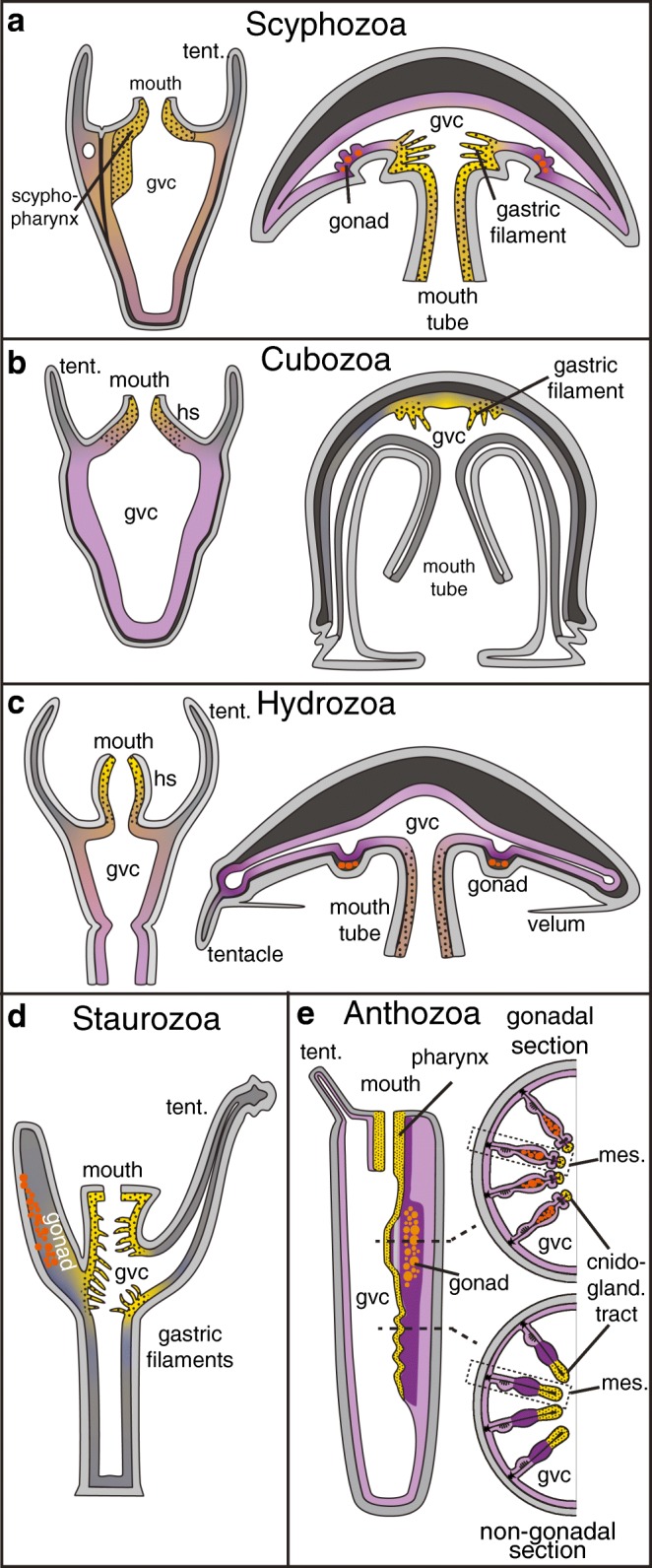
**a**–**d** The distribution of tissues with extra- or intracellular digestion in scyphozoan (a), cubozoan (b), hydrozoan (c), staurozoan (d) and anthozoan (e) cnidarians. Dotted yellow: exclusively exocrine; purple: exclusively phagocytic/pinocytic; brown: mixed tissues. Grey: no exocrine, phagocytic or pinocytic activity described. Boxed regions in (**e**) are magnified in Fig. 4a and b. In staurozoans and cubomedusae, no data is available on phagocytic or pinocytic tissues or cell types. Gvc: gastrovascular cavity; hs: hypostome; mes: mesentery; tent: tentacle

All cnidarians are composed of two epithelial sheets: the outer epidermis and inner gastrodermis with an intermediate, jelly-like extracellular matrix (mesoglea) (Fig. 3). Polyps and medusae possess a single body opening, which functions as both mouth and anus. It opens into a gastrovascular cavity (GVC), which serves as both digestive and circulatory space (Fig. 3). The mouth opening and GVC form during gastrulation, at planula larva stages, or during metamorphosis to the polyp stage, depending on the gastrulation type (Byrum and Martindale 2004; Tardent 1978). In anthozoans and scyphozoans, of which most gastrulate by invagination, the endoderm forms as thickened epithelium during early gastrulation and invaginates as an epithelial sheet (Byrum and Martindale 2004; Tardent 1978). The blastopore gives directly rise to the mouth opening (Byrum and Martindale 2004; Tardent 1978). Endoderm was widely thought to give rise to all of the gastrodermis throughout cnidarians, but as will be described in a later section, this has recently been refuted in a sea anemone (Brusca et al. 2016; Martindale et al. 2004; Steinmetz et al. 2017). Hydrozoans and cubozoans gastrulate predominantly by ingression or delamination of cells into the blastocoel until it is completely filled up. The GVC and mouth opening, and thus the inner digestive tissues, typically form only at planula stages or during metamorphosis into polyps (Byrum and Martindale 2004; Tardent 1978).

Digestion in cnidarians is generally a two-step process: first, prey is broken up in the gastrovascular cavity by digestive enzymes secreted from exocrine gland cells (often termed ‘zymogen’ cells in the literature); then, food particles and nutrients are distributed throughout the GVC, and phago- or pinocytosed into gastrodermal cells where digestion is finalised by lysosomal enzyme activities (Fautin and Mariscal 1991; Lesh-Laurie and Suchy 1991; Thomas and Edwards 1991; Van-Praët 1985). Historically, the term ‘zymogen cell’ has been used for gland cells secreting extracellular digestive enzymes in cnidarians and bilaterians, mainly based on histochemical similarities with pancreatic zymogen cells (Tiffon 1975; Van Praët 1978; Van-Praët 1985). The term zymogen implies that secreted digestive enzymes act as proenzymes, which need processing by other enzymes to function as hydrolase. Currently, however, there is no direct biochemical evidence to support this claim in any cnidarian.

In medusae, a mouth tube leads to a digestive ‘stomach’, from which a more or less intricate system of gastrovascular canals distributes food particles throughout the body (Arai 1997; Bouillon et al. 2006; Thomas and Edwards 1991). A main difference between medusae of different groups is the presence and location of specialised ‘gastric cirri/filaments’. They are found in cubozoan and scyphozoan medusae, and in the ‘stalked medusae’ of staurozoans (Fig. 3a–d) (Arai 1997; Lesh-Laurie and Suchy 1991). Wherever present, gastric cirri reach into the central stomach, but are localised at its oral side (scyphozoans), aboral side (cubozoans) or on both sides (staurozoans) (Fig. 3a–d). They often contain a variety of distinct zymogen cells and cnidocytes (Arai 1997; Di Camillo et al. 2006; Heeger and Möller 1987; Miranda et al. 2013) and are thus similar in structure and function to the cnidoglandular tract at the tip of gastrodermal outfolds as described below for anthozoan polyps (Van-Praët 1985) (see below).

*In situ* hybridisation studies found *trypsin*, *chitinase* and *pancreatic lipase* genes expressed in zymogen-like cells along gastric cirri of young *Aurelia aurita* (Scyphozoa) medusae (Steinmetz et al. 2017). A recent transcriptomic analysis of the cirri in the cubozoan *Alatina* has revealed an over-representation of toxin-like genes (e.g. *conotoxin-like* genes) and digestive enzymes (*chymotrypsin*-like genes), suggesting a dual role of this tissue in extracellular digestion and venom release (Lewis Ames et al. 2016). Other body regions of cubomedusae also participate in extracellular digestion. Extracellular proteases are also secreted from the stomach wall, but the underlying cell types are currently unknown (Fig. 3b) (Larson 1976). In hydrozoan (which lack cirri) and scyphozoan medusae, zymogen cells are also found in high concentrations along the gastrodermis of the oral tube and mouth arms (Fig. 3a, c) (Arai 1997; Bouillon et al. 2006; Steinmetz et al. 2017).

Phagocytosis of food particles and intracellular digestion has been described throughout the gastrovascular cavities of scyphomedusae and hydromedusae (Fig. 3a, c) (Arai 1997; Bouillon et al. 2006; Hyman 1940). An increased phagocytic activity is seen in gastrodermal epitheliomuscular cells of the mouth tube and somatic gonad regions of both *Aurelia* and *Clytia*, and in tentacle bulbs of *Clytia* (Amiel et al. 2010; Bouillon et al. 2006). Knowledge of phago- or pinocytic cells in cubomedusae and stauromedusae is currently lacking.

Polyps of different cnidarian groups differ in the degree of compartmentalisation and specialisation of the gastrodermis. Hydrozoan and cubozoan polyps both lack any inner subdivision. Hydrozoans include the relatively well-studied research model species *Hydra*, *Hydractinia, Podocoryne* and *Clytia*. In most hydrozoan polyps, zymogen cells are predominantly found in the gastrodermis of the mouth region (hypostome) (Fig. 3c). Phagocytic and intracellular digestive activities are in contrast predominantly found in epitheliomuscular cells of the median body column (Fig. 3c) (Bouillon et al. 2006). In colonial hydrozoans, the gastrodermis of stolons, the basal connections between individual polyps, further distributes and phagocytoses food particles (Bouillon et al. 2006). The cnidarian research model *Hydra* lacks a medusa stage and coloniality, and has an atypical distribution of ‘digestive’ cell types: zymogen cells are intermingled among phagocytic cells in the gastrodermis of the mid-gastric region, but not the hypostome, where instead mucous cells are enriched (Haynes and Davis 1969; Rose and Burnett 1968). Notably, cell lineage studies in *Hydra* suggest that midbody zymogen cells trans-differentiate into oral mucous cells (Siebert et al. 2008).

Cubopolyps are relatively poorly studied, except for *Tripedalia cystophora*. Similar to most hydrozoans, potential zymogen cells in *Tripedalia* are found predominantly in the oral gastrodermis (‘oral cone’) (Fig. 3b). Phagocytic cells are found more abundantly in aboral regions, where they make up to 80% of all cells (Fig. 3b) (Chapman 1978; Lesh-Laurie and Suchy 1991).

Anthozoan and scyphozoan polyps, in contrast to hydro- and cubopolyps, show a variable number of gastrodermal folds (termed ‘septa’ or ‘mesenteries’) that subdivide the GVC into inter-connected compartments (Fig. 3e). Scyphozoan polyps (‘scyphistoma’) possess four, tetrameric septa reaching into the GVC (Lesh-Laurie and Suchy 1991), while anthozoan septa (or mesenteries) are organised as pairs along a bilateral-symmetrical ‘directive’ axis (Berking 2007). All septa are characterised by a higher concentration of zymogen cells at their distal tip (Fig. 3a, e).

Only little is known on the distribution and function of digestive cell types in scyphistoma. Zymogen cells, potentially secreting chitinases or proteases, are concentrated in the ‘scypopharynx’ and septae of *Aurelia* polyps, but their presence elsewhere in the gastroderm remains unclear (Fig. 3a) (Arai 1997; Chia et al. 1984; Heeger and Möller 1987; Hyman 1940). In *Aurelia*, a contact between prey and septa appears essential for digestion to occur, suggesting that digestive enzymes are not freely diffusing within the GVC (Bumann and Kuzirian 1996). Studies in a number of scyphopolyps revealed that phagocytic or pinocytic activities are found throughout the gastrodermis, including the septa and pharynx (Arai 1997; Blanquet and Wetzel 1975; Chia et al. 1984; Fitt and Trench 1983; Heeger and Möller 1987). Notably, several studies have supported a major ectodermal contribution to the gastrodermis of the *Aurelia* scyphistoma (Gold et al. 2016; Mayorova et al. 2012; Yuan et al. 2008).

### Origin and cell type diversity of anthozoan digestive tissues

**Fig. 4:**
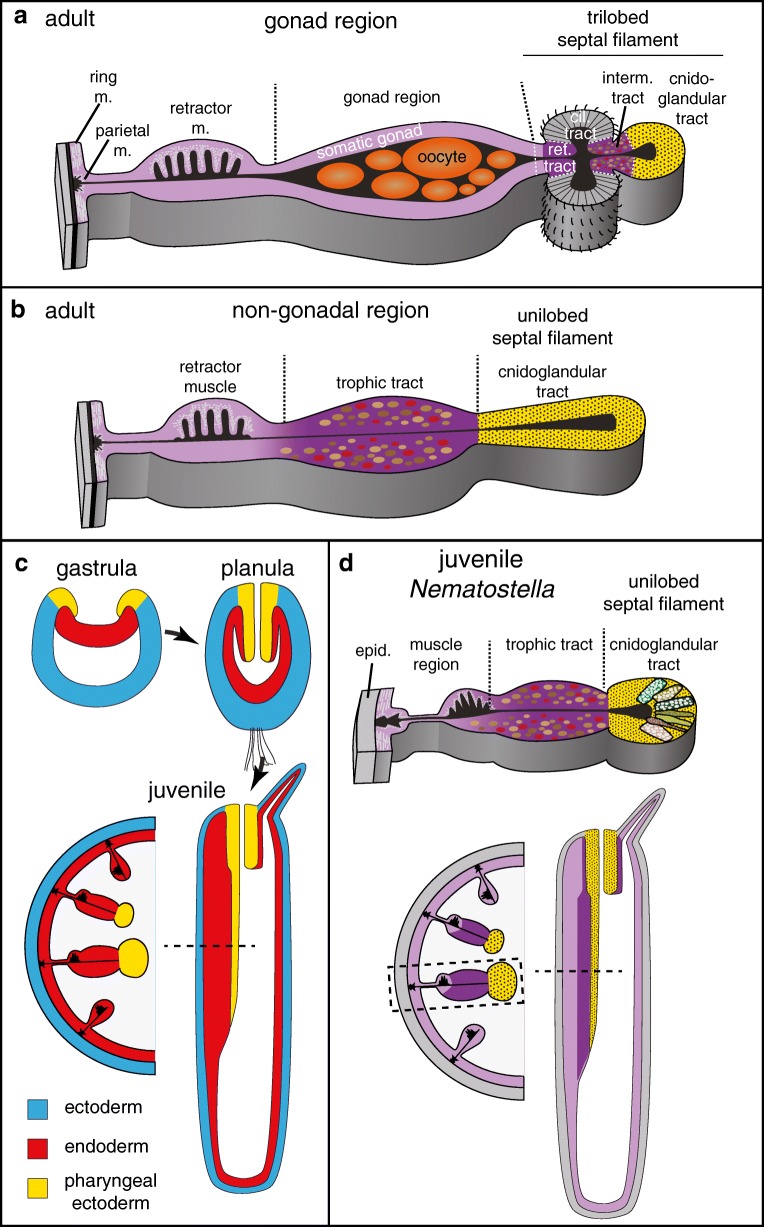
Development, structure and cell type composition of adult (**a**, **b**) or juvenile (**c**, **d**) mesenteries of the sea anemone *Nematostella vectensis*. **a**, **b** Schematic cross-section of adult mesenteries at gonadal (**a**) and non-gonadal (**b**) levels. **c** Schematic development and fate map from gastrula stages until juvenile polyp. All schematics represent longitudinal cross-sections except lower right (cross section). **d** Schematic representation of exocrine (dotted yellow) and phagocytic (purple) tissues in juvenile Nematostella polyps. Upper and lower left schematics are cross-sections while lower right schematics is a longitudinal section. Cil: ciliated; epid.: epidermis; m. muscle; ret.: reticulate; interm.: intermediate

Digestive processes have been relatively well studied in anthozoans, where the main digestive tissues are localised in the pharynx and the mesentery, an outfold of the inner gastrodermis (Fig. 3e). The mesenterial structure is best described for sea anemones, where it is subdivided into a basal muscular, a distal septal filament region and a median part that, at variable positions along the oral-aboral axis, harbours the gonad (Figs. 3e and 4a, b) (Fautin and Mariscal 1991; Shick 1991; Van-Praët 1985). Gametes develop within the extracellular matrix (‘mesoglea’) in-between the two epithelial sheets of the mesentery. The adult distal septal filament of sea anemones has either a ‘unilobed’ or ‘trilobed’ shape (Fig. 4a, b). Their distribution is variable among anthozoan groups, and in sea anemones, both types typically co-occur in one animal (Daly et al. 2003).

The unilobed and tip of the trilobed septal filaments have essential roles in extracellular digestion (Fautin and Mariscal 1991). Their cell type composition is very similar to the pharynx, containing cnidocytes and zymogen cells (Fautin and Mariscal 1991). This part of the mesentery is therefore termed ‘cnidoglandular tract’ (Fig. 4a, b). Physiological studies show that these tissues produce chitinase- and trypsin-like enzymes (Nicol 1959; Van-Praët 1985). It has been observed that digestive enzyme activities are low in the GVC, and that mesenteries need to ‘wrap’ the prey for efficient digestion (Nicol 1959). This led to the proposal that, as in the scyphistoma, extracellular digestion is contact-dependent. This suggests two possibilities for the localisation of digestive enzymes: either they are secreted and concentrated within the mucus layer (similar to the bilaterian ‘brush border’ enzymes), or they are tethered to the membrane. Domain structure analysis of digestive enzymes in *Nematostella* suggests that both possibilities may occur in sea anemones: chitinases, lipases and a subset of trypsin-domain proteins are mostly single domain proteins without transmembrane domains; some trypsin domains co-occur together with extracellular protein-protein interaction MAM domains, which can bind to MAM domain-containing membrane receptors (Beckmann and Bork 1993; Steinmetz et al. 2017). Cells of the cnidoglandular tract are not phagocytic, but have high amino acid uptake capacities (Van Praët 1980; Van-Praët 1985). It is currently unclear if zymogen cells are functionally similar to vertebrate/insect enterocytes. While their secretory function is obvious, further research needs to clarify if zymogen cells are also capable of amino acid uptake, and if this amino acid uptake process is based on a transmembrane transport system similar to enterocytes. It is also completely unclear if septal filament cells can potentially export amino acids via basolateral transporters into the mesoglea, from where they could reach other body parts.

Tissues responsible for extra- and intracellular digestion appear strictly separated in sea anemones (Fig. 4a–d). Phagocytosis can occur throughout the gastrodermis, with the exception of the cnidoglandular tract and the ‘ciliated tract’ of the trilobed septal filament (Fig. 4a–d). Certain regions, however, appear to show increased phagocytic uptake of larger food particles (Fig. 4a) (Van-Praët 1985). The ‘intermediate tract’, linking the cnidoglandular and ciliated tracts, exhibits high phagocytic activity. It consists of mono-ciliated cells with microvilli (Fig. 4a) (Van-Praët 1985). A water current generated by the neighbouring ciliated tract supposedly traps particles in the groove of the intermediate tract, where they are phagocytosed. In sea anemones with symbionts, zooxanthellae are abundant in the intermediate tract as well as the tentacular and oral disc gastrodermis (Van-Praët 1985). Also, the region between the median gonad region and ciliated tract, the ‘reticulated tract’, shows increased phagocytic and pinocytic activities (Fig. 4a) (Van-Praët 1985). It is currently unclear if the somatic gonad epithelium surrounding the developing gametes has any specific role in phago- or pinocytosis. Feeding of radio-labelled amino acids has revealed that a specialised cell population within the somatic gonad, the ‘trophonema’, might play a role in translocating nutrients from the GVC into the oocyte (Eckelbarger et al. 2008; Larkman and Carter 1982). This assumption is yet to be studied on a molecular level. In non-gonadal parts of the mesentery, the median region of mesenteries ('trophic tract') shows high phagocytic activities (Fig. 4b) (Van-Praët 1985). The concentration of phagocytosis or amino acid uptake to some parts of the animal raises the question if under fasting conditions nutrients are transported from these regions to more remote parts of the sea anemone body via the GVC or possibly even the mesoglea.

In order to understand how exocrine and phagocytic cells of sea anemones compare to the bilaterian gut cell types, it is necessary to combine the available ultrastructural and physiological data with molecular and developmental data. So far, digestive tissues of anthozoans have only been studied thoroughly on these levels in juveniles of the sea anemone *Nematostella vectensis*. In contrast to adults, mesenteries of juvenile *Nematostella* are almost entirely consisting of the unilobed type. It therefore remains speculative if they mostly resemble the non-gonadal parts of the adult mesentery. Double *in situ* hybridisation studies and single-cell transcriptomic analyses have identified a high diversity of exocrine cells in the pharynx and cnidoglandular tract, including three distinct *trypsin-*, two different *pancreatic lipase-* and one *chitinase*-expressing zymogen cells (Sebé-Pedrós et al. 2018b; Steinmetz et al. 2017). Their discovery supports previous physiological studies and reveals a higher diversity of zymogen cells than previously defined by ultrastructural studies (Frank and Bleakney 1976; Shick 1991; Van-Praët 1985). In *Nematostella*, the expression of genes encoding for secreted digestive enzymes, and thus reflecting the location of potential zymogen cells, is strictly restricted to the pharynx and cnidoglandular tract. These tissues also harbour insulinergic gland cells among others cell types yet to be characterised (Sebé-Pedrós et al. 2018b; Steinmetz et al. 2017). In both *Nematostella* and *Aurelia* (see above), the combination of exocrine and insulinergic cells is confined to tissues expressing the *foxA* transcription factor, which is a conserved marker of bilaterian endoderm, midgut or vertebrate foregut (Sebé-Pedrós et al. 2018b; Steinmetz et al. 2017). This combination of exocrine and insulinergic cells within a *foxA+* tissue is thus reminiscent of the bilaterian midgut, and especially the vertebrate pancreas. Previous studies, based on histochemical and physiological methods, came to a very similar conclusion (Tiffon 1975; Van Praët 1978; Van-Praët 1985).

### Exocrine pharynx and cnidoglandular tracts develop from ectoderm

A cell lineage analysis in *Nematostella* has revealed that the juvenile cnidoglandular tract—despite its ‘gastrodermal’ location—develops from cells originating in the larval pharyngeal ectoderm, and not the endoderm (Fig. 4c) (Steinmetz et al. 2017). A common developmental origin of pharynx and cnidoglandular tract is also reflected by the shared composition of exocrine, insulinergic and cnidocyte cells. While an ectodermal origin of the pharynx of sea anemones and soft corals (Octocorallia) has been widely accepted, studies from the nineteenth and early twentieth century had also already speculated about an ectodermal origin of the cnidoglandular tract in soft corals (Octocorallia) (Ax 1996; Chia and Crawford 1973; Matthews 1916; Tardent 1978; Wilson 1883; Wilson 1884). Together, these data suggest that their ectodermal origin is ancestral to anthozoans (Wilson 1884). Notably, a major part of the gastrodermis of the scyphozoan polyp and the exocrine pharynx of ctenophores are also derived from ectoderm, which makes it possible that an ectodermal origin of exocrine tissue even dates back prior to the last common ancestor of cnidarians and bilaterians (Gold et al. 2016; Mayorova et al. 2012; Yuan et al. 2008).

All remaining gastrodermal tissues of *Nematostella* juveniles develop from the endodermal germ layer (Fig. 4c) (Steinmetz et al. 2017). This includes the median mesenterial region, which was previously named ‘somatic gonad’, and which has been shown to store glucose and lipids in juvenile *Nematostella* (Steinmetz et al. 2017). Its strong pinkish colour in fed juveniles (from *Artemia* brine shrimps) implies that this region has a generally increased phagocytic activity of food particles. As juveniles have no obvious gonad, and as it is not clear if this median region will contribute to the adult gonad epithelium, I propose to re-name this juvenile region ‘trophic tract’ (Fig. 4c).

Paradoxically, as described above, the cell composition (zymogen and insulinergic) and physiological functions (digestive enzyme secretion and amino acid uptake) of *Nematostella* ectoderm derivatives is similar to the endodermally derived bilaterian midgut (and especially the vertebrate pancreas). These similarities are further corroborated on a molecular level: *Nematostella* orthologs of genes with conserved expression in bilaterian midgut tissues (*foxA*, *hex*) and vertebrate pancreas development (*nkx2.2*, *tbx2/3*, *islet*, *nkx6*, *hlxb9*, *soxB1*) consistently co-localise to the larval pharyngeal ectoderm of *Nematostella* (Steinmetz et al. 2017). The juvenile trophic tract does not show co-localisation of any of these genes. Instead, this phagocytic tissue co-expresses *Nematostella* orthologs of *foxC*, *six4/5* and *nkx3/bagpipe* transcription factors, whose bilaterian counter-parts are consistently co-localised to bilaterian visceral mesoderm (Sebé-Pedrós et al. 2018b; Steinmetz et al. 2017). A large number of other *Nematostella* transcription factors, whose bilaterian orthologs have important roles in mesoderm specification or development (e.g. *tbx* genes, *hand*, *twist*, *mox*), have almost exclusively been found expressed in different endodermally derived parts of the *Nematostella* gastrodermis (e.g. cardiac muscle-like parietal and circular muscles)(Martindale et al. 2004; Ryan et al. 2007; Steinmetz et al. 2017). Transcription factor profiles have thus further confirmed the similarities between bilaterian endodermal midgut (and vertebrate pancreas) and the pharyngeal ectoderm of *Nematostella*. Sea anemone endoderm, in contrast, shows strong similarities to bilaterian mesoderm but not endoderm. Due to the lack of cell lineage and transcription factor expression data from adult *Nematostella*, the developmental origin of adult-specific tissues, such as gonads or parts of the trilobed mesenterial filament, is currently unclear.

### A new scenario of germ layer evolution and its consequences for the evolution of digestive systems

**Fig. 5:**
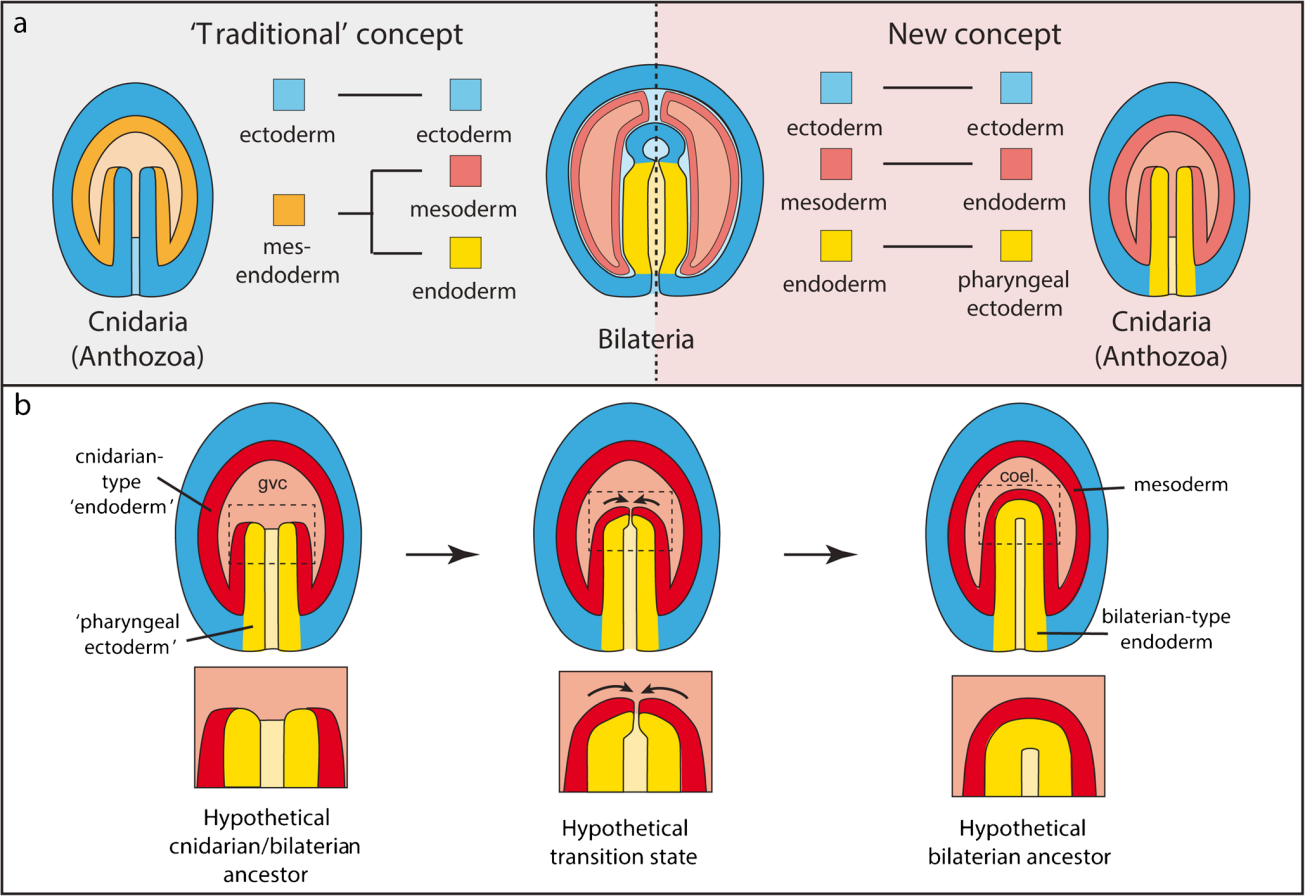
Schematic representations of germ layer homologies between cnidarians and bilaterians (**a**) and a hypothetical evolutionary scenario of the transition between two-layered (diplobastic) and three-layered (triploblastic) animals (**b**). Regions within dashed boxes in (b) are magnified below. coel.: coelomic cavity; gvc: gastrovascular cavity

The finding that pharyngeal ectoderm, and not endoderm of sea anemones, is most similar to bilaterian endoderm has important consequences for our understanding of bilaterian germ layer and body plan evolution, including the evolution of digestive systems. It refutes the idea at the core of Haeckel’s gastraea theory (Fig. 5a, ‘traditional concept’) that bilaterian and cnidarian endoderm germ layer derivatives have similar function or share a common origin (Haeckel 1873). Instead, it strongly supports an alternative model (Fig. 5a, ‘new concept’) of germ layer homologies between sea anemones (and thus cnidarians) and bilaterians, where:

- The pharyngeal ectoderm, developing near the blastopore margin, is homologous to bilaterian endoderm
- The sea anemone endoderm is not homologous to both bilaterian endoderm and mesoderm, as previously suggested (Byrum and Martindale 2004; Martindale et al. 2004).
- The sea anemone endoderm is only homologous to bilaterian mesoderm.

This model supports the notion that all three bilaterian germ layers were topologically separate in the last common ancestor of cnidarians and bilaterians (Fig. 5b). Bilaterian mesoderm would thus not be an evolutionary novel tissue but correspond to the cnidarian-type endoderm of the last common ancestor. The bilaterian novelty would only have consisted in the changing location to an intermediate position between ectoderm and endoderm, as typical for bilaterians. How can we imagine that this transition has occurred? Based on a scenario by E.B. Wilson, I present here a simple and plausible evolutionary transition to explain the formation of both the bilaterian gut as well as the intermediate mesoderm from a sea anemone-like ancestral situation (Fig. 5b) (Wilson 1884). I propose that the crucial change during early bilaterian evolution was the fusion of the distal-most part of the tube-like pharyngeal ectoderm, which opens into the GVC in sea anemones. This would have resulted in the formation of an exocrine, blind-ended digestive sac corresponding to a primitive bilaterian gut (Fig. 5b). Notably, such a blind-ended epithelial gut is proposed to be ancestral for xenacoelomporphs, which form the sister group to all remaining bilaterians (Hejnol and Pang 2016). As an immediate consequence, the ancestral endoderm would become positioned in-between the developing gut (the ‘new’ bilaterian endoderm) and the outer ectoderm and would thus lie in the typical position of the bilaterian mesoderm (Fig. 5b).

This scenario makes a number of testable predictions:

- The gastrula fate map of sea anemones is ancestral for cnidarians. Exocrine tissue should thus be expected to originate from oral ectoderm in other cnidarian groups, especially the ones gastrulating by invagination (e.g. scypohozoans) (Byrum and Martindale 2004). More fate maps from gastrulae of different cnidarian groups should allow testing this hypothesis.
- One of the ancestral functions of the mesoderm was phagocytosis. As a consequence of the mesoderm adopting its intermediate location, and losing contact with the external environment, its phagocytic functions became largely dispensable. Interestingly, phagocytic immune cells residing in the blood system of coelomic cavities, such as vertebrate macrophages (Herbomel et al. 1999), sea urchin (Smith et al. 2006) or annelid (Vetvicka and Sima 2009) coelomocytes or *Drosophila* hemocytes (Holz et al. 2003; Tepass et al. 1994) are all developing from mesoderm. They might thus represent evolutionary relicts of the phagocytic function of ancestral cnidarian-type endoderm derivatives. Notably, secondary mesenchyme cells of sea urchins show phagocytic activity from mid-gastrula stage onwards (Silva 2000). Also, nutrient-storing trophic cells, such as annelids eleocytes (Vetvicka and Sima 2009) or the insect fat body cells (Moore et al. 1998; Riechmann et al. 1998) develop from mesoderm in bilaterians and might reflect an ancestral function of mesoderm in nutrient storage similar to the ‘trophic region’ of sea anemones. A better molecular characterisation and expression profile comparison between cnidarian and bilaterian phagocytic cells (including gut phagocytes) will help resolving their evolutionary relationship (see also Hartenstein et al. in this issue).
- Bilaterian coelomic pouches and cnidarian gastrovascular pouches, subdivided by mesenteries, are directly homologous, as previously proposed by the so-called enterocoely concept (Arendt 2004; Remane 1950; Sedgwick 1884; Tautz 2004). This prediction would require endodermal subdivisions into pouch-like segments being ancestral for cnidarians and bilaterians. A recent study suggests that the ancestral function of Hox genes in the common ancestor of cnidarians and bilaterians might have been to position the boundaries of such subdivisions (He et al. 2018).

The further evolutionary transition from a blind gut with one opening to the typical through-gut of bilaterians with a mouth and anus has been a subject of intense debate, and out of the scope of this review (Hejnol and Martindale 2009; Nielsen et al. 2018).

### Early animal ‘digestive cavities’ probably had functions in phagocytosis, but not extracellular digestion

The gastrodermis of sea anemones, scyphozoans and ctenophores is derived from endoderm, and secondarily internalised pharyngeal ectoderm. As it would make most sense to secrete digestive enzymes into a digestive cavity, it sounds counter-intuitive that exocrine cells are specified within the outer ectoderm in these animals. In ‘design terms’, a direct specification and differentiation of exocrine cells in the inner endodermal germ layer would have been much more straightforward. The peculiar development of cnidarian-type endoderm and exocrine cells might therefore reflect their evolutionary history, and it can be speculated that:

- The primary role for the evolution of cnidarian-type endoderm was phagocytosis, and not the secretion of digestive enzymes. It supports the notion that digestive cavities were primarily sites for endo- and not extracellular digestion, similar to the choanocyte chambers in sponges.
- Due to the consistent development of cnidarian-type endoderm into epitheliomuscular cells and gonad tissue, the primary reason(s) for its evolution were:
  - The protection of the germline
  - Enhancement of contractile and motile functionalities
  - Formation of the primary, apical-blastoporal body axis with a functional specialisation of the substrate-facing side in phagocytosis. This would resemble the situation also in *Trichoplax*, where the ventral epithelial cells play a major role in pinocytosis (see above)
- Extracellular digestion has evolved after the appearance of the phagocytic cavity. The specification of exocrine cells within the ectoderm near the blastopore margin suggests an ancestral digestive or protective (defence against pathogens) function of this region, as partly proposed previously in the ‘mucociliary sole’ concept (Arendt et al. 2015).

## Conclusion

Altogether, recent data is in line with Haeckel’s assumption that the sea anemone endoderm reflects an ancient absorptive cavity. Future studies will potentially resolve if the cell types deriving from cnidarian endoderm derivatives are similar in their gene expression profiles and functional capacities to the cell types found in the ctenophore gastrovascular system, the endocytic lower epithelium of placozoans, or the phagocytic choanocytes or archaeocytes. The core of the gastraea theory, consisting of the idea that the bilaterian gut evolved from the endoderm of a cnidarian-like ancestor, is however not supported by recent data. I have put forward an alternative theory of germ layer and gut homology between cnidarians and bilaterians that can be put to the test in future studies.
